# Efficacy of Cognitive Behavior Therapy, Pelvic Floor Therapy, Integrated Therapy, and Pharmacotherapy in the Treatment of Chronic Pelvic Pain in Women: A Randomized Controlled Trial

**DOI:** 10.12669/pjms.42.9.16890

**Published:** 2026-09

**Authors:** Bushra Akram, Ahmad Bilal

**Affiliations:** 1 Bushra Akram, Department of Applied Psychology, The Islamia University of Bahawalpur, Bahawalpur, Pakistan; 2 Ahmad Bilal, Department of Applied Psychology, The Islamia University of Bahawalpur, Bahawalpur, Pakistan

**Keywords:** Chronic Pelvic Pain, Cognitive Behavior Therapy, Pelvic Floor Therapy, Integrated Therapy, Randomized Controlled Trial

## Abstract

**Objective:**

The objective of the current study was to determine the efficacy of Cognitive Behavior Therapy (CBT-cp), Pelvic Floor Therapy (PFT), Integrated Therapy (Combination of CBT-cp and PFT), and Pharmacotherapy in the Treatment of Chronic Pelvic Pain in women, compared with the placebo group.

**Methodology:**

A five-arm (four intervention arms and one placebo arm), Randomized Controlled Trial (RCT) was conducted, to examine the effectiveness of CBT-cp, PFT, and Pharmacotherapy in the treatment of Chronic Pelvic Pain in women, compared with the placebo group. The sample comprised 175 female patients with CPP; 141 patients completed the study. They were presented to the Outpatient Department of the Department of Obstetrics & Gynecology, and the Department of Physiotherapy at Nishtar Hospital, Multan, Pakistan, from April to June, 2025. The Impact of Female Chronic Pelvic Pain Questionnaire (IF-CPPQ), Urdu version was used as primary outcome measure. The intervention lasted three months.

**Results:**

Results showed that the mean CPP scores significantly decreased at the Posttest (*p* < 0.001) on IF-CPPQ, although they did not maintain at follow-up. It demonstrated that posttest and follow-up scores were significantly different from pretest scores in all the intervention groups. The Integrated therapy group differed considerably from the CBT-cp, PFT, Pharmacotherapy, and Placebo groups in CPP reduction.

**Conclusion:**

The Integrated therapy is the most effective and statistically significant in reducing CPP compared with CBT-cp, PFT, Pharmacotherapy, and Placebo.

***Registration No.:*** ClinicalTrials.gov (Identifier: NCT07042555).

## INTRODUCTION

Chronic Pelvic Pain (CPP) is persistent pain in the pelvis, lasting at least six months without a clear etiology.^1^ The pain may be chronic and incapacitating, dull or sharp, cramping or of a pressure-like nature, and constant or intermittent. Chronic Pelvic Pain in women is a real condition that is experienced frequently. The prevalence of CPP in women is from 5.7% to 26.6%. The reason for pain in up to 70% of CPP patients is not gynecological. In Pakistan, the prevalence is 15.75%, and 93% have never visited a pain specialist.^2^,^3^

The etiology of CPP and its predisposing factors are not well known. Patients who experience CPP are reluctant to talk about it with their friends, family, and loved ones since it impacts the most personal areas of human life, such as sexuality and reproduction. Chronic Pelvic Pain involves psychological, gastrointestinal, gynecological, musculoskeletal, urological, dysfunctional sexuality, and behavioral factors. Pakistani women with unknown organic etiology are a complex issue with many possible explanations.^4^

Psychological factors have a significant impact on CPP. Chronic Pelvic Pain mainly involves physical problems, such as myofascial pelvic floor dysfunction, and psychological concerns like negative beliefs, worry, stress, anxiety, and depression. Patients with CPP typically have a poor prognosis. Most patients with pelvic pain are treatable, and they should be given dignity and respect in addition to being heard. Moreover, medical health professionals are likely to ignore the symptoms and consider them to be psychological or psychiatric disorders.^5^,^6^

Worldwide, healthcare settings now prioritize chronic pelvic pain management, and caregivers can manage it if they identify the source of pain. A multidisciplinary approach is essential to the assessment, diagnosis, and management of patients with CPP. Cognitive Behavior Therapy for Chronic Pain (CBT-cp) is the most effective treatment for CPP. As a goal-directed psychotherapy, CBT-cp helps patients to identify negative thoughts, beliefs, and behaviors about CPP and alter them. After changing unhelpful thought patterns, patients can promote more adaptable coping strategies. It reduces the intensity of CPP and its symptoms, also improving the quality of life and daily functioning.^7^, ^8^

Pelvic Floor Therapy (PFT) is also a valuable treatment and plays an essential role in the management of CPP. There are different types of exercises to improve muscular coordination, strength, relax muscles, and decrease pain sensitivity. In Pakistan, the majority of the patients are unaware of the role of PFT in managing CPP. The hurdles include: lack of knowledge, shortage of specialists, expensive interventions, inadequate healthcare facilities, shame and stigma around the issues, lack of referrals, limited research and education about PFT.^9^-^10^

In CPP, a health care professional can prescribe a variety of medicines. Proper and adequate pharmacological management for CPP has a significant effect, but it is short-term. In pharmacotherapy, pain relievers such as Anticonvulsants, Nonsteroidal Anti-inflammatory Drugs (NSAIDs), Antidepressants, Muscle relaxants, and opioids are mainly prescribed depending on the cause.^11^

A new, innovative, and more effective approach for CPP is Integrated therapy (Combined CBT-cp and PFT). In the integrated approach, psychological techniques like CBT-cp to control pain perception, stress, anxiety, and emotional response linked to CPP, and PFT for muscle problems, help to strengthen pelvic floor muscles and reduce CPP. When combined, they break the cycle of pain, stress, and fear and enhance healing. Some researchers recommend integrated management because single therapies typically do not work.^12^,^13^

The study objective was to determine the efficacy of Cognitive Behavior Therapy, Pelvic Floor Therapy, Integrated Therapy, and Pharmacotherapy in the treatment of Chronic Pelvic Pain in women, compared with the placebo group. We hypothesized that Cognitive Behavior Therapy for Chronic Pain, Pelvic Floor Therapy, Integrated Therapy, and Pharmacotherapy are effective in the treatment of Chronic Pelvic Pain in women, compared with the placebo group. In Pakistan, there is lack of evidence on the efficacy of psychotherapeutic and integrated treatment approaches in the treatment of CPP in women. The current Randomized Controlled Trial fills this gap.

## METHODOLOGY

It was a Randomized Controlled Trial (RCT) conducted at Department of Obstetrics & Gynecology, and the Department of Physiotherapy at Nishtar Hospital, Multan, Pakistan, from April to June, 2025 to examine the effectiveness of CBT-cp, PFT, Integrated Therapy (CBT-cp combined with PFT), and Pharmacotherapy in the Treatment of Chronic Pelvic Pain in women, compared with a Placebo group. It was a five-arm study, with four Intervention arms and one Placebo arm.

### Ethical approval:

After ethical approval from the Departmental Research Committee (DRC) (5612/A.Psy, dated: April 24, 2024), and the Advanced Studies and Research Board (AS&RB) of the Islamia University of Bahawalpur, the study was registered on ClinicalTrials.gov (Identifier: NCT07042555). In the intervention groups, all patients were informed about the intervention throughout the study. All patients signed informed consent. Participants were also informed about the study’s benefits, procedures, and duration and had the right to withdraw from the study at any time. Trained professionals administered each intervention. Participants were informed that the interventions had minimal adverse effects; CBT-cp may cause emotional distress; PFT can create temporary physical discomfort; and pharmacotherapy could have adverse effects such as burning, stomach upset, constipation, dizziness, and nausea. During the trial, any adverse effects or participant concerns were addressed and handled in accordance with the study protocol. No serious side effects had been reported in any intervention, except for upset stomach in pharmacotherapy. The Gynecologist also prescribed medicine to treat stomach issues caused by medications. In the CBT-cp, PFT, and Integrated Therapy Intervention groups, it was not possible to blind participants and therapists due to the nature of the behavioral and physical therapy techniques. In the Pharmacotherapy and Placebo groups, single-blinding was used. To maintain blinding, the Placebo medications are designed to be equivalent in duration, and frequency, and form. Confidentiality was strictly maintained throughout the study. Any participant had the right to refuse to participate in the study.

### Instruments

After signing informed consent, the demographic Form was filled out. It was designed to collect information regarding age, education, locality, marital status, occupation, site of pain, pain duration, and pain severity, and previous and current medications. To assess CPP in women, the Impact of Female Chronic Pelvic Pain Questionnaire (IF-CPPQ), Urdu version was used. It is a 26-item, self-reported questionnaire, that uses five-point likert scale, and takes around 5 to 10 minutes to complete. It is a five-factor scale that includes: Psychological Impact, Emotional Impact, Sexual Impact, Relationship Impact, and Occupational Impact. The minimum score is 0, and the maximum score is 104.^14^

### Participants

The study population consisted of women aged 18-46 years who presented to the Departments of Obstetrics & Gynecology and Physiotherapy at Nishtar Hospital, Multan, Pakistan, with CPP. The sample comprised of 175 female patients with CPP; 34 patients dropped out, and 141 patients completed the study. G*Power was used to calculate the 80% power, alpha *α*=0.05, effect size *f*=0.25 (medium, based on previous CPP trials), and 20% attrition, *n*=31/arm minimum, rounded to 35 for subgroups.

### Participants recruitment and allocation:

Participants were recruited through random allocation to the Interventions groups and the Placebo group. A Randomization method was used to minimize bias and ensure equal distribution of patients across the four Intervention groups and one Placebo group. Randomization was performed using the Research Randomizer (http://www.randomizer.org/).^15^ Each participant had an equal chance of being assigned to any of the five groups because the random sequence list was created before the participants’ allocation into groups. The Allocation Concealment technique was also used to prevent selection bias for assigning patients to the four intervention groups (CBT-cp, PFT, Integrated Therapy Pharmacotherapy) and one placebo group. A sequential assignment process based on the pre-generated randomization list was used to preserve allocation concealment. Until randomization, it concealed the assignment sequence from participants and researchers. A 1:1:1:1:1 allocation ratio was used to assign participants randomly. Participants’ recruitment in the Intervention and the Placebo groups is shown in the flowchart in Fig.1.

**Fig.1:**
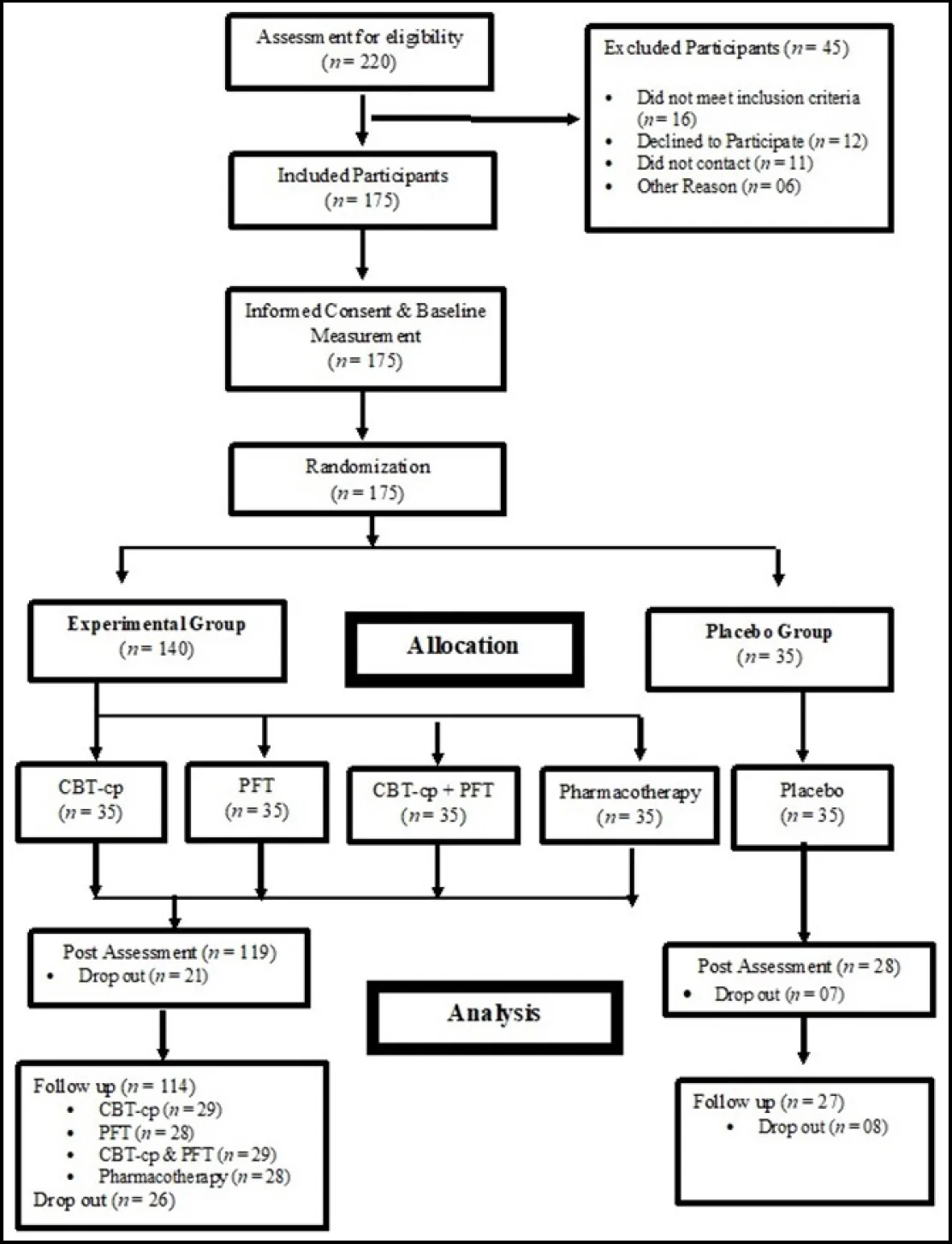
This CONSORT diagram shows patient recruitment.

### Inclusion and exclusion criteria are as follows:

### Inclusion criteria:

- Female patients aged 18-46 years, women with provoked Vestibulodynia (PVD), women with Bladder Pain Syndrome (BPS), and women with CPP without organic pathology, were included in the study.

### Exclusion criteria:

- Women with peri-menopause or with menopause, pregnant women, any organic gynecological illness, medical or surgical cause, active gynecological infection, women with psychiatric disorder, and women with substance use were not included in the study.

### Attrition rate:

In this study, 28 (16%) patients dropped out of the posttest assessment, and 34(19.4%) dropped out of the follow-up assessment. The reasons for dropout are: declined to participate, dissatisfaction with the intervention, and chronicity itself.

### Primary outcome measure:

The primary outcome measure was the IF-CPPQ (Urdu version), which assessed the intensity of CPP, its impact on daily functioning, and the effectiveness of interventions for CPP in women.

### Procedure

The study was conducted between April and June, 2025. Patients with CPP initially presented to the Outpatient Department of Obstetrics & Gynecology and Physiotherapy at Nishtar Hospital, Multan, Pakistan. A Gynecologist did a baseline assessment and diagnosed CPP. They referred women with CPP who met the eligibility criteria to a Psychotherapist for CBT-cp, to a Physiotherapist for PFT, and to both experts for Integrated therapy. The Gynecologist prescribed Pharmacological Therapy and a Placebo. Participants were selected through randomization and a homogeneous sample; predefined inclusion and exclusion criteria were followed, and the same assessment procedure for the outcome measure was used in the posttest and follow-up to control confounding variables. Weekly sessions, intervention compliance, visit schedules, pain severity, homework assignments, pre-assessment, post-assessment, and follow-up schedules were followed by the researchers. Pretest, Posttest, and follow-up assessment were done using IF-CPPQ. A follow-up assessment was conducted three months after the termination of interventions, in September 2025.

### Interventions

In the CBT-cp group, patients participated in 12 weekly individual sessions lasting 50-60 minutes each. The Psychological intervention was delivered by the Principal Researcher, who is a Principal Clinical Psychologist (BS-19), following a comprehensive training course at The Beck Institute of Cognitive Behavior Therapy in Bala Cynwyd, Pennsylvania, United States. The session format of CBT-cp techniques included: Psychological Assessment, Cognitive Restructuring about pain, Thought stopping, CBT Techniques (Exposure, Graded Task Assignments, Pacing, Activity Scheduling, Distraction techniques, Progressive Muscle Relaxation, Stress Management, Pain coping skills, Problem Solving), Rehearsal and Application of the techniques, and review of Homework assignments. The researcher also monitored compliance, briefed patients on the benefits of the intervention to increase motivation and progress, reviewed homework assignments, reviewed the session notes and checklist for intervention reliability and follow-up schedules, and addressed all patient queries.

In the PFT group, six sessions of 30-60 minutes were conducted over six weeks, performed by an experienced, and in-practice Physiotherapist. The format of the PFT sessions included an Initial Assessment, Correct Muscle Identification, Kegel exercise (sometimes with additional exercises such as squats, bridges, or core-strengthening movements), Progression, Integration, and review of Homework Assignments. During therapy, all protocols and considerations for exercise were also provided (e.g., stretches, 10-15 repetitions, three to five seconds holding, three to five seconds rest).

The Gynecologist prescribed medicines to patients in pharmacotherapy group. The patients in the control group were given placebo by the gynecologist. The Pharmacotherapy included NSAIDs Naproxen Sodium 550mg, OD for 15-30 days; Cyclobenzaprine 5mg titrated to 10mg OD for 15-30 days, Amitryptaline 25mg for three months, and Gabapentin 100mg titrated to 200mg OD for three months. The medicines were adjusted as per the requirements of the patients and intensity of the symptoms. The medicines were tapered off before discontinuation. The Gynecologist also prescribed Proton Pump Inhibitors (PPIs) (Omeprazole 40mg, once daily/if needed) to treat stomach issues caused by medications. In the Placebo group, as in pharmacotherapy, the dose was adjusted according to the severity of symptoms.

### Statistical analysis:

Descriptive statistics, and Repeated Measures Analysis of Covariance (RM-ANCOVA) were conducted using the Statistical Package for the Social Sciences (SPSS) version 27. Results were shown in the tables.

## RESULTS

The frequency distribution of the demographic variables included in the study is shown in Table-I. There were 141 participants in the study: 29 in the CBT group, 28 in the PFT group, 29 in the Integrated therapy group, 28 in the Pharmacotherapy group, and 27 in the Placebo group. Groups were similar in all demographic characteristics.

**Table-I:** Frequency Distribution of Demographic Variables (N=141)

Therapy Groups	CBT Therapy (n=29)		PFT Therapy (n=28)		Integrated Therapy (n=29)		Pharmacotherapy (n=28)		Placebo Therapy (n=27)	
Demographic Variables	F	%	F	%	F	%	F	%	F	%
***Age Groups***										
18-20	5	17.24	6	21.42	5	17.24	4	14.28	5	18.51
21-30	13	44.82	10	35.72	15	51.72	13	46.42	11	40.75
31-40	8	27.59	10	35.72	7	24.14	5	17.85	9	33.34
41-46	3	10.35	2	7.14	2	6.90	6	21.42	2	7.40
***Locality***										
Urban	21	72.42	22	78.58	17	58.62	21	75.0	17	62.96
Rural	8	27.58	6	21.42	12	41.38	7	25.0	10	37.04
***Education***										
Matric or Below	7	24.13	10	35.71	7	24.13	9	32.14	14	51.85
Inter	5	17.24	6	21.43	6	20.69	3	10.71	3	11.11
Bachelors	8	27.59	6	21.43	6	20.69	6	21.42	3	11.11
Masters / MPhil/PhD	9	31.04	6	21.43	10	34.49	10	35.71	7	25.93
***Occupation***										
Student	6	20.69	4	14.28	4	13.79	4	14.28	4	14.82
House Wife	12	41.37	15	53.58	18	62.07	14	50.0	18	66.66
Working Women	11	37.94	9	32.14	7	24.14	10	35.71	5	18.52
***Marital Status***										
Single	9	31.04	7	25.0	8	27.58	8	28.57	8	29.63
Married	18	62.06	18	64.29	18	62.07	17	60.72	17	62.97
Divorced / Widow	2	6.90	3	10.71	3	10.35	3	10.71	2	7.40
***No of Children***										
N/A	9	31.03	8	28.57	8	27.59	8	28.58	8	29.63
Issueless	2	6.90	3	10.72	4	13.80	4	14.28	3	11.11
1-2	11	37.94	10	35.71	12	41.37	11	39.28	8	29.63
3-5	7	24.13	7	25.0	5	17.24	5	17.86	8	29.63
***Method of Delivery***										
N/A	11	37.93	11	39.28	12	41.37	11	39.28	11	40.74
Operation	5	17.24	4	14.29	4	13.80	3	10.72	9	33.33
SVD	13	44.83	13	46.43	13	44.83	14	50.0	7	25.93
***Site of Pain***										
Lower Abdomen	17	58.63	18	64.28	18	62.06	18	64.28	21	77.78
Pelvic Bone	12	41.37	10	35.72	11	37.94	10	35.72	6	22.22
***Duration of Pain***										
6 months-1year	17	58.63	18	64.28	17	58.63	10	35.72	8	29.63
2-4 years	9	31.03	7	25.0	9	31.03	7	25.0	13	48.14
5-6 years	3	10.34	3	10.72	3	10.34	11	39.28	6	22.23
10+ years										
***Pain Intensity***										
Mild	7	24.13	7	25.0	8	27.58	9	32.14	7	25.93
Discomforting	11	37.94	9	32.14	11	37.94	11	39.28	12	44.44
Distressing	11	37.93	12	42.86	10	34.48	8	28.58	8	29.63
***Previous Treatment***										
No Treatment	16	55.17	19	67.86	12	41.37	18	64.28	16	59.25
Pain Killers	13	44.83	9	32.14	17	58.63	10	35.72	11	40.75
***Current Treatment***										
Yes	5	17.24	6	21.42	5	17.24	8	28.58	5	18.52
No	24	82.76	22	78.58	24	82.76	20	71.42	22	81.48

The descriptive measures of the therapy groups of the study. CBT-cp, PFT, Integrated Therapy, Pharmacotherapy, and Placebo therapy groups had 29, 28, 29, and 27 participants, respectively. Mean scores on CPP significantly decreased at the Posttest, but were not maintained at follow-up. Despite lower scores in the CBT-cp, PFT, and Integrated Therapy groups compared with the Pharmacotherapy and Placebo group, both groups increased to near pretest levels in the follow-up, shown in Table-II.

**Table-II:** Descriptive Statistics of Therapy Groups at Pretest, Posttest, and Follow-up (N=141).

Pre Test		
Variables	Chronic Pelvic Pain	N
Treatment Groups	M (SD)	
CBT	44.14 (12.15)	29
PFT	43.86 (13.90)	28
Integrated Therapy	42.79 (11.55)	29
Pharmacotherapy	42.43 (12.98)	28
Placebo group	44.96 (8.92)	27
***Post Test***		
***Variables***	***Chronic Pelvic Pain***	***N***
Treatment Groups	M (SD)	
CBT	16.31 (4.85)	29
PFT	20.96 (11.59)	28
Integrated Therapy	10.97 (3.41)	29
Pharmacotherapy	28.75 (13.03)	28
Placebo group	30.44 (12.88)	27
***Follow Up***		
***Variables***	***Chronic Pelvic Pain***	***N***
Treatment Groups	M (SD)	
CBT	19.72 (7.47)	29
PFT	24.43 (10.78)	28
Integrated Therapy	13.00 (3.50)	29
Pharmacotherapy	35.54 (16.11)	28
Placebo group	38.74 (10.58)	27

The within-subjects pairwise comparisons of the significance of differences in mean CPP scores at pretest, Posttest, and follow-up across all intervention groups is shown n Table-III. It is shown that posttest and follow-up scores were significantly different from pretest scores in all the intervention groups. The posttest and follow-up scores were also significantly different.

**Table-III:** Within-Subjects Pairwise Comparisons (N=141).

(I) factor1	(J) factor1	Mean Difference (I-J)	SE	p	95% Confidence Interval	
					Lower Bound	Upper Bound
***CBT Therapy Group***						
1	2	27.82	2.12	0.000	23.46	32.18
	3	24.41	2.23	0.000	19.82	29.00
2	1	-27.82	2.12	0.000	-32.18	-23.46
	3	-3.41	.71	0.000	-4.88	-1.93
3	1	-24.41	2.23	0.000	-29.00	-19.82
	2	3.41	.71	0.000	1.93	4.88
***PFT Therapy Group***						
1	2	22.89	2.12	0.000	18.53	27.25
	3	19.42	2.41	0.000	14.47	24.38
2	1	-22.89	2.12	0.000	-27.25	-18.53
	3	-3.46	1.39	0.020	-6.33	-.59
3	1	-19.42	2.41	0.000	-24.38	-14.47
	2	3.46	1.39	0.020	.59	6.33
***Integrated Therapy Group***						
1	2	31.82	1.96	0.000	27.79	35.86
	3	29.79	1.99	0.000	25.69	33.89
2	1	-31.82	1.96	0.000	-35.86	-27.79
	3	-2.03	.46	0.000	-2.98	-1.08
3	1	-29.79	1.99	0.000	-33.89	-25.69
	2	2.03	.46	0.000	1.08	2.98
***Pharmacotherapy Group***						
1	2	13.67	2.00	0.000	9.55	17.80
	3	6.89	1.46	0.000	3.88	9.90
2	1	-13.67	2.00	0.000	-17.80	-9.55
	3	-6.78	1.81	0.001	-10.50	-3.06
3	1	-6.89	1.46	0.000	-9.90	-3.88
	2	6.78	1.81	0.001	3.06	10.50
***Placebo Group***						
1	2	14.51	1.79	0.000	10.83	18.20
	3	6.22	1.38	0.000	3.37	9.07
2	1	-14.51	1.79	0.000	-18.20	-10.83
	3	-8.29	1.36	0.000	-11.11	-5.48
3	1	-6.22	1.38	0.000	-9.07	-3.37
	2	8.29	1.36	0.000	5.48	11.11

***Note:*** 1=Pretest, 2=Posttest, 3=Follow up.

Four intervention groups and one placebo group in terms of reductions in CPP scores are shown in Table-IV. The Integrated therapy group differed significantly from the PFT, Pharmacotherapy, and Placebo groups in pain reduction. On the other hand, CBT-cp was not significantly different from Integrated Therapy. The CBT-cp group showed greater pain reduction than the Pharmacotherapy and Placebo groups, whereas there was no significant difference in pain reduction between the CBT-cp and PFT groups. The PFT group differed considerably from the Pharmacotherapy and Placebo groups, including the CBT-cp group. The Pharmacotherapy and Placebo groups did not differ significantly in pain reduction. It shows that Pharmacotherapy did not show a statistically significant benefit over the placebo and was less effective than other non-pharmacological interventions.

**Table-IV:** Between-Subjects Pairwise Comparisons (N=141)

Chronic Pelvic Pain Scores						
(I) Treatment	(J) Treatment	Mean Difference (I-J)	SE	p	95% Confidence Interval	
					Lower Bound	Upper Bound
CBT	PFT	-2.76	2.30	0.23	-7.32	1.80
	Integrated Therapy	4.02	2.28	0.08	-.50	8.54
	Pharmacotherapy	-8.23	2.31	0.001	-12.80	-3.66
	Placebo	-11.41	2.32	0.000	-16.01	-6.80
PFT	CBT	2.76	2.30	.23	-1.80	7.32
	Integrated Therapy	6.78	2.31	0.004	2.20	11.35
	Pharmacotherapy	-5.47	2.32	0.020	-10.07	-.86
	Placebo	-8.65	2.35	0.000	-13.29	-4.00
Integrated Therapy	CBT	-4.02	2.28	0.08	-8.54	.50
	PFT	-6.78	2.31	0.004	-11.35	-2.20
	Pharmacotherapy	-12.25	2.31	0.000	-16.83	-7.67
	Placebo	-15.43	2.33	0.000	-20.04	-10.82
Pharmaco-therapy	CBT	8.23	2.31	0.001	3.66	12.80
	PFT	5.47	2.32	0.020	.86	10.07
	Integrated Therapy	12.25	2.31	0.000	7.67	16.83
	Placebo	-3.17	2.35	0.17	-7.83	1.47
Placebo	CBT	11.41	2.32	0.000	6.80	16.01
	PFT	8.65	2.35	0.000	4.00	13.29
	Integrated Therapy	15.43	2.33	0.000	10.82	20.04
	Pharmacotherapy	3.17	2.35	0.17	-1.47	7.83

## DISCUSSION

The demographic information in this study is consistent with those in other studies. The subjects in the other studies were all females with CPP, 60% were married, and 66% had a secondary school education,^16^ CPP affects women of reproductive age,^3^ 23% of the subjects were from rural areas, 22% from urban areas, and 9% had a higher CPP in rural areas.^17^ In an urban environment, patients prefer medical help for CPP because of the availability of health care facilities. The characteristic is also consistent with epidemiological studies on CPP, which indicate that young and middle-aged women are most affected by this disorder and that it has a high prevalence in women of reproductive age.^3^,^8^

In this study, patients with the highest percentage, 70 (49.65%), had CPP for 6 months to one year, 92 (65.25%) had lower abdominal pain, and 54 (38.30%) to 49 (34.75%) had a discomforting to distressing level (Moderate to Severe) of CPP intensity. It aligns with research highlighting the severity of symptoms that require a structured intervention.^18^ A study conducted on patients with CPP, 81 (54.45%) had never received treatment for CPP, and 112 (79.43%) were currently not receiving treatment.^19^The findings of the current research are consistent with the outcomes of the systematic review and meta-analysis, which indicated that CPP remained under-treated and that delayed diagnosis was a frequent occurrence that could have led to self-medication with over-the-counter painkillers.^13^ In Pakistani culture, especially in South Punjab and among patients from rural areas, many patients’ expectations are for strong and multiple medications for the effective treatment of pain. It was a significant challenge to improve compliance with non-pharmacological interventions. Inadequate knowledge of the long-term advantages of Interventions, cultural beliefs, embarrassment related to pelvic health issues, and low health literacy all further impair adherence.

The descriptive statistics for this study’s therapy groups at pretest, posttest, and follow-up (Table-II) showed a significant improvement in CPP from pretest to posttest across all groups; however, the improvement was not stable at follow-up. In posttest assessment, there was a reduction in pain score in the CBT-cp group (*M* = 16.31, *SD* = 4.85), the PFT group (*M* = 20.96, *SD* = 11.59), the pharmacotherapy group (*M* = 28.75, *SD* = 13.03), and the placebo group (*M* = 30.44, *SD* =12.88). The integrated therapy group showed the most significant reduction in pain scores (*M* = 10.97, *SD* = 3.41). All groups showed a minor increase in pain levels at the follow-up assessment. It indicates a minor decrease in improvement over time. In the integrated therapy group, the mean CPP score dropped from 42.79 at pretest to 10.97 in the posttest and 13.00 at follow-up. This research supports the biopsychosocial and multidisciplinary interventions. The Integrated therapy, which offers significant and prolonged pain relief to patients with CPP, is used in clinical practice and research. Other researchers showed that CBT-cp focuses on psychological and behavioral elements of CPP, and it helps in reducing the perception of pain, distress, and disability. It has a significant impact on reducing pain.^8^,^20^An RCT feasibility pilot study concluded that CBT-cp is an effective non-pharmacological intervention for CPP reduction in women. Participants in the CBT-cp intervention group reported significant post-therapy improvement in pain intensity, whereas the Control group showed no change.^9^ Other studies concluded that the Integrated Therapy produced the best and most effective improvements. Pharmacological interventions often provide only short-term relief; however, they are not as lasting as the behavioral and integrated interventions that address the biopsychosocial factors of CPP. The CBT-cp and integrated interventions are more effective than a single approach to CPP.^12^,^21^ In a RCT on a mindfulness-based stress reduction treatment, contrary to this study, no statistically significant between-group differences were observed.^22^ Pakistani-specific cultural and health care systems may also influence the level of therapy response. Patients in semi-urban and rural areas sometimes have limited knowledge of PFT and CBT-cp. Furthermore, due to limited knowledge, cultural norms, practical difficulties, and the need to visit multiple times for CBT and PFT in both government hospital departments, unaffordability in a private setting, and adherence to at-home PFT may be suboptimal. These factors may explain discrepancies between this study’s results and research conducted in developed countries with well-established, comprehensive CPP treatments.

The findings of this study show significant effects on managing CPP in the pretest, posttest, and follow-up evaluations, as evidenced by within-subjects pairwise comparisons (Table-3) and between-subjects pairwise comparisons (Table 4). The mean difference in pain scores between the pretest and posttest was statistically significant (*p* < 0.000), indicating that all interventions produced a measurable change across all groups. Posttest and follow-up differences across all groups were also significant (p ≤ 0.020), with a negative mean difference indicating that pain scores increased slightly from posttest to follow-up. However, the difference between the pretest and follow-up was large and significant (*p* < 0.000), indicating that despite some decline, all therapies maintained a considerable overall benefit compared to baseline. The integrated therapy group produced the most significant pain reduction compared to placebo (Mean difference = 15.43, *p* < 0.001), and both CBT-cp group and PFT group as a standalone intervention (Mean difference = 11.41, *p* <0.001), and (Mean difference = 8.65, *p* < 0.001) respectively improved the CPP. At the same time, Pharmacotherapy did not significantly differ from Placebo (Mean Difference = 3.17, *p* < 0.05). The results support the Integrated Therapy and CBT-cp for the management of CPP. Other studies also concluded that the long-lasting behavioral effects of integrated interventions demonstrate their benefits. It is a superior intervention compared to single interventions such as PFT or pharmacotherapy. Pharmacotherapy works best when combined with behavioral and physical therapies.^23^-^25^ Two systematic reviews and a meta-analysis of non-pharmacological treatments for CPP in women were conducted, using multidisciplinary methods, including PFT with CBT. The findings of both the systematic review and the meta-analysis were contrary to this study, which concluded that physical therapy had the strongest evidence; in contrast, psychological techniques, acupuncture, and other non-pharmacological treatments had less or insufficient evidence and were insignificant.^13^ The 2^nd^ research also demonstrated that non-pharmacological treatments improved quality of life and pain alleviation. Evidence confidence, however, was very low. These findings emphasize the need for further thorough research to validate the effectiveness of non-pharmacological treatments in pain management.^18^

In this study, although the Placebo group showed a reduction in pain in the posttest (*M* = 30.44) (Table-III), it was significantly less than the experimental groups. It did not maintain and increased to near pretest levels in the follow-up. It indicates that although patients may improve subjective symptoms for a short time, or reduce pain perception, the underlying cause remains. In this study, follow-up was conducted three months after the initial assessment. A longer follow-up is required to evaluate loss of impact over time, as the three month follow-up period was very short; thus, any early improvements might not accurately reflect the durability of the benefit. There was single-blinding in the placebo group, which might affect participant expectations and reporting of results, and is challenging to avoid in behavioral and physical therapy interventions. Attrition reduced the final sample size available for analysis, potentially introducing bias and reducing statistical power.

### Implications

The research findings have direct clinical implications. It provides valuable insight into the psychological and behavioral approaches to CPP management in addition to pharmacotherapy. The standalone pharmacological interventions often involve higher risks and adverse side effects. Multidisciplinary management is an effective alternative to stand alone pharmacotherapy for management of CPP. It is the first RCT study to evaluate the CBT-cp and Integrated therapy as first-line treatment for CPP.

### Limitations

The study population consisted only of women with CPP, limiting generalizability for men. A follow-up was conducted three months after treatment; it was brief and insufficient to determine the long-term durability of these therapies. Single-blinding was used in the pharmacotherapy and placebo groups, but not in the CBT-cp, PFT and Integrated groups, and performance bias might have affected the results.

## CONCLUSION

The results of this study strongly support the integration of psychological treatment with pharmacotherapy in the treatment of Chronic Pelvic Pain in women, though, CBT-cp can also give effective results as compared to pharmacotherapy alone. It concluded that the Integrated therapy and CBT-cp were the most effective and statistically significant modalities in reducing CPP in comparison with PFT, Pharmacotherapy, and Placebo.

### Recommendations

In future studies, men with CPP should be included for broader generalizability. A longitudinal study with long-term follow-up, such as six months or one year, can better evaluate the long-term efficacy of interventions. To minimize performance bias, outcome measures and follow-up should be conducted by double blinded assessors in further research.

### Author’s Contribution:

**BA:** Conceptualization, Investigation, Project Administration, Data Curation, Formal Analysis, Writing-Original Draft. **AB:** Conceptualization, Methodology, Writing-Reviewing, Editing, Supervision and responsible for accuracy of the study.
